# Amino acid residues that are important for Hyal2 function as a receptor for jaagsiekte sheep retrovirus

**DOI:** 10.1186/1742-4690-2-59

**Published:** 2005-09-28

**Authors:** Fuh-Mei Duh, Clarissa Dirks, Michael I Lerman, A Dusty Miller

**Affiliations:** 1 Basic Research Program, SAIC-Frederick, National Cancer Institute at Frederick, Frederick, Maryland 21702, USA; 2 Laboratory of Immunobiology, Center for Cancer Research, National Cancer Institute at Frederick, Frederick, Maryland 21702, USA; 3 Fred Hutchinson Cancer Research Center, Seattle, Washington 98109, USA; 4 Current address: University of Washington, Seattle, Washington 98195, USA

## Abstract

**Background:**

Infection by jaagsiekte sheep retrovirus (JSRV) and by enzootic nasal tumor virus (ENTV) depends on cell-surface expression of the virus entry receptor, hyaluronidase 2 (Hyal2). Human Hyal2 binds the envelope (Env) proteins of these viruses and is functional as a receptor, but Hyal2 from mice does not bind Env nor does it mediate entry of either virus. Here we have explored the amino acid determinants that account for the difference in receptor function.

**Results:**

Analysis of human-mouse Hyal2 chimeric proteins showed that amino acid differences responsible for the difference in Hyal2 receptor activity were localized to the central third of Hyal2. Human Hyal2 mutants containing single or double amino acid replacements with the respective mouse amino acids were generated across this region and were assayed for activity. None of the single or double mutation reduced the receptor activity of human Hyal2 by more than 10-fold, whereas mouse Hyal2 activity is reduced 1,000-fold from that of human Hyal2. While the 3-dimensional structures of mammalian Hyal2 proteins are unknown, bee venom hyaluronidase shows significant amino acid similarity to human and mouse Hyal2 and its structure has been determined. Many mutations having the largest negative effects on human Hyal2 function mapped to a small region of the bee venom hyaluronidase close to but not overlapping the active site of the enzyme, suggesting that this site represents the binding site for Env. Analysis of synonymous and non-synonymous nucleotide substitutions in the coding sequences of multiple mammalian Hyal2 proteins shows that the proteins are undergoing strong selection for amino acid conservation. We found no evidence for positive selection of amino acid changes that might reflect evolution of mammalian hosts to resist JSRV or ENTV infection.

**Conclusion:**

These results show that the greatly reduced receptor activity of mouse Hyal2 in comparison to that of human Hyal2 is determined by multiple amino acid changes acting in concert. In particular, no one amino acid change blocks infection. However, the most important amino acids map to a small patch on a predicted 3-dimensional Hyal2 structure, which may represent the binding site for Env.

## Background

JSRV and ENTV are closely-related retroviruses that induce tumors in the lower airways and nasal epithelium, respectively, of sheep and goats [1]. Both viruses utilize the glycosylphosphatidylinositol-anchored cell-surface protein Hyal2 as a receptor for cell entry [2]. Hyal2 is a member of a family of proteins, some of which exhibit high hyaluronidase activity and are capable of rapid degradation of hyaluronan, a component of the extracellular matrix. However, Hyal2 exhibits only weak hyaluronidase activity [3] and its primary biological role in mammals is not known. Evidence for Hyal2 function as the virus receptor is provided by experiments showing that expression of sheep or human Hyal2 in cells that are not normally susceptible to infection renders the cells fully infectable by retroviral vectors bearing the JSRV or ENTV Env proteins [4-7]. For example, the titers of JSRV and ENTV vectors on mouse cells expressing human Hyal2 are >1,000-fold higher than those on cells expressing mouse Hyal2. In addition, the receptor-binding surface (SU) domains of the JSRV and ENTV envelope proteins bind with high affinity to cells expressing human Hyal2 but not to cells expressing nonfunctional receptors such as mouse Hyal2 [6,8,9], indicating that Hyal2 is the primary receptor that binds virus prior to entry.

Here we have used a transient transfection assay to analyze the amino acid differences between human and mouse Hyal2 that account for the large difference in receptor activity of the two proteins. Non-susceptible NIH 3T3 mouse cells were transfected with the Hyal2 expression constructs and receptor function was quantitated by measuring transduction of the cells with a JSRV vector. We could localize most of the difference in receptor function to the central 38% of the Hyal2 proteins. Single and double amino acid changes made throughout this region in human Hyal2 to convert the residues to those in the mouse sequence were made and tested. None of the changes reduced human Hyal2 activity more than 10-fold, but a combination of three of the most important mutations reduced the activity by 17-fold. We conclude that the difference in receptor activity between human and mouse Hyal2 is explained by the effects of multiple amino acid changes acting together. However, the most important amino acids mapped to a small surface region of the known bee venom hyaluronidase suggesting that this is the binding site for Env.

## Results

### Deletions of the amino or carboxy termini of human Hyal2 abrogate receptor activity

**Figure 1:**
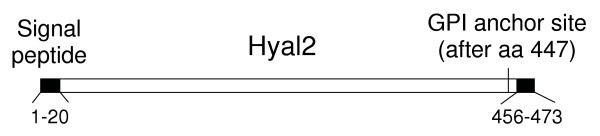
**Human Hyal2 protein features**. Amino acids 1–20 constitute the endoplasmic reticulum signal peptide for human Hyal2 [3]. A glycosylphosphatidylinositol (GPI) anchor is predicted to replace all residues following amino acid 447 in human Hyal2, which contains a C-terminal hydrophobic tail at residues 456–473 that localizes the protein in the membrane prior to GPI anchor addition.

The overall structure of human Hyal2 is shown in Fig. 1. The protein is directed to the endoplasmic reticulum by a 20 amino-acid signal peptide at the amino terminus [3], deletion of which abolishes the receptor activity of human Hyal2 [5]. However, this signal can be replaced with a preprotrypsin signal sequence followed by a Flag peptide tag without affecting the receptor activity of human Hyal2 [5]. To determine if portions of the amino terminus beyond the signal sequence could be deleted without affecting receptor activity of human Hyal2, we made a series of deletion mutants that consisted of the preprotrypsin signal sequence followed by the Flag tag fused in frame to the remainder of the protein. Deletion of amino acids 1–30 of human Hyal2 (corresponding to a deletion of 10 amino acids after the signal peptide) reduced its activity to 1% of that of human Hyal2, and deletions of 40, 50, 60, 120, 180, 240, and 300 amino-terminal amino acids reduced receptor activity to undetectable levels (data not shown). Loss of receptor activity could be due to an inability of the JSRV Env to bind the truncated receptors, to defects in later steps of virus entry mediated by Hyal2, or to improper folding or processing of the deletion mutant Hyal2 proteins to the cell surface. Regardless, these data show that most of the amino terminus of Hyal2 is required for proper receptor function, with the possible exception that a subset of the first 10 amino acids after the signal peptide may be dispensable for receptor function.

The carboxy end of Hyal2 is predicted to contain a GPI addition signal, and experimental data confirms the presence of the GPI linkage of Hyal2 to the cell surface [5]. Complete removal of the GPI addition signal (amino acids 440–473) or only the GPI addition site (amino acids 440–453) abrogated the receptor activity of human Hyal2 [5]. We have not explored whether internal deletions upstream of the GPI signal are compatible with receptor function or whether other membrane linkages might yield functional receptors, but it is clear that the carboxy end of Hyal2 is important for normal receptor function.

### Analysis of chimeric mouse/human Hyal2 proteins localizes amino acids responsible for the difference in receptor activity to the central region of the proteins

**Figure 2:**
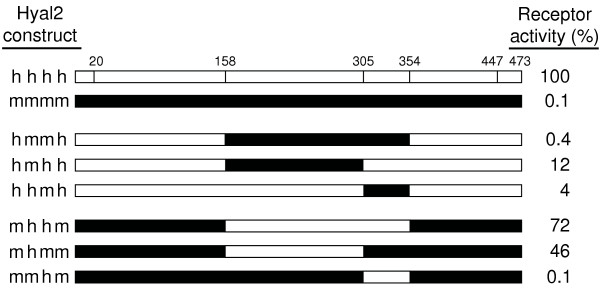
**Receptor activity of mouse/human receptor chimeras**. Scale drawings of human (open boxes), C3H mouse (black boxes) and chimeric Hyal2 receptors are shown. Receptor activities following transfection of NIH 3T3 mouse cells are indicated at right and are expressed as a percentage of that of human Hyal2. The top diagram shows the positions of the signal peptide (amino acids 1–20), the GPI anchor addition site (after amino acid 447), and the amino acid positions where the Hyal2 cDNAs were recombined. The human Hyal2 [GenBank:U09577.1] and C3H mouse Hyal2 [GenBank:AF302843.1] sequences used here have been described. The Hyal2 protein sequence of C3H mice is identical to that of NIH Swiss mice [GenBank:AF535140.1], and differs by one amino acid (V at position 355) from that of Czech II mice [GenBank:AF302844.1] (I at position 355).

Mouse and human Hyal2 are 82% identical at the amino acid level but mouse Hyal2 shows 1,000-fold lower JSRV receptor activity. In an attempt to localize the amino acids responsible for this difference we made a series of human/mouse Hyal2 chimeras and assayed them for receptor function (Fig. 2). Hyal2 was divided into four domains based on the availability of convenient restriction enzyme sites in the cDNAs at positions corresponding to amino acids 158, 305, and 354. Constructs are named based on the order of the mouse and human sequences. Construct hmmh has very low activity while construct mhhm has activity near that of human Hyal2, showing that the central regions of mouse and human Hyal2 are responsible for most of their receptor phenotype. Constructs hmhh and hhmh have activity between that of mouse and human Hyal2 showing that both the second and third domains of mouse Hyal2 contribute to the low activity of mouse Hyal2. Constructs mhmm and mmhm show that insertion of the second domain of human Hyal2 can restore most of the activity of mouse Hyal2, while insertion of the third domain of human Hyal2 is unable to increase the low activity of mouse Hyal2. In conclusion, differences between mouse and human Hyal2 in the central regions of these proteins are primarily responsible for large difference in JSRV receptor activity of the two proteins.

### Analysis of human Hyal2 mutants containing single and double mouse amino acid substitutions shows cooperative effects of the changes

**Figure 3:**
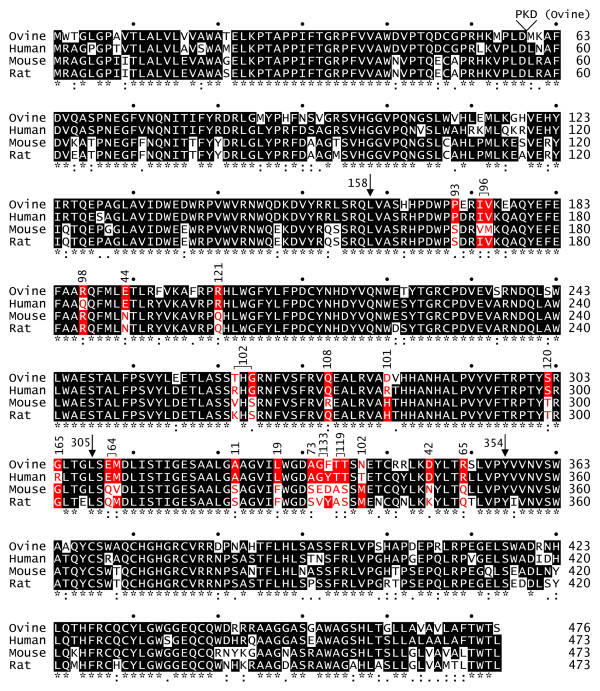
**Alignment of Hyal2 protein sequences from different species and effect on receptor function of conversion of amino acid residues in human Hyal2 to those present in mouse Hyal2**. ClustalW [23] was used to generate an alignment of ovine [GenBank:AF411974.1], human [GenBank:U09577.1], C3H mouse [GenBank:AF302843.1], and rat [GenBank:AF535141.1] Hyal2 protein sequences. Ovine Hyal2 has a three amino acid insertion following amino acid 56 that is not present in the other Hyal2 proteins. Amino acid identity (*), strong similarity (:), weaker similarity (.), and dissimilarity (no mark) are indicated below the sequences. Downward arrows indicate positions of junctions between chimeric human/mouse Hyal2 chimeras, and dots above the sequences indicate 10 base-pair intervals. Residues that differ between human and mouse Hyal2 in the region between amino acids 158 and 354 are indicated in red, and the receptor activities (% of human Hyal2 activity) of human Hyal2 proteins containing the single or double replacements with the respective mouse amino acids are shown above the amino acid residues. Where two amino acids were altered, a bracket depicts the residues that were altered.

To identify the individual mouse amino acid changes that account for the decreased activity of mouse Hyal2, we made human Hyal2 mutants containing single and double mouse amino acid substitutions throughout the critical central domain defined above (Fig. 3). Most changes had little effect on receptor activity, but one in the second domain (E189N, 44% activity) and two in the third domain (A322S, 11% activity; L327F, 19% activity) had relatively low activities. Importantly, no one amino acid replacement reduced the activity of human Hyal2 by more than 10-fold, whereas mouse Hyal2 activity is reduced 1,000-fold from that of human Hyal2. We also made a human Hyal2 mutant that contained all three of these mutations, and this mutant had 6% of the activity of human Hyal2 (average of two experiments, data not shown). We conclude from analysis of these human Hyal2 mutants and from the mouse/human chimeric Hyal2 results that the low JSRV receptor activity of mouse Hyal2 is due to the combined action of multiple amino acid changes acting in concert.

Interestingly, the mutation R301G increased the receptor activity of human Hyal2 by 65%. A glycine is present at this position in both the mouse and the sheep receptor. The sheep receptor functions better as a JSRV receptor than does the human receptor in previous assays [6], suggesting that this glycine is important to achieve the highest receptor activity.

We also made several changes to mouse Hyal2 to see if its activity could be increased by replacement of mouse amino acids with the corresponding amino acids from human Hyal2. Based on the results above, we made the mutations F327L, N189E, and both mutations together in mouse Hyal2. All of these mutants had receptor activities (0.2% of human Hyal2, means of 2 experiments for each construct) similar to mouse Hyal2 (0.1%). The result for the N189E mutation is particularly interesting since replacement of the second domain of mouse Hyal2 with that of human Hyal2 restores nearly full receptor activity (construct mhmm in Fig. 2), and none of the human Hyal2 mutants containing single and double replacements with the corresponding mouse amino acids significantly reduced human Hyal2 activity besides the E189N replacement (Fig. 3). Again, these results indicate that alteration of receptor activity is due to multiple amino acid changes acting in concert.

### Mouse Hyal2 accumulates at the cell surface at levels at least as high as those of human Hyal2

**Figure 4:**
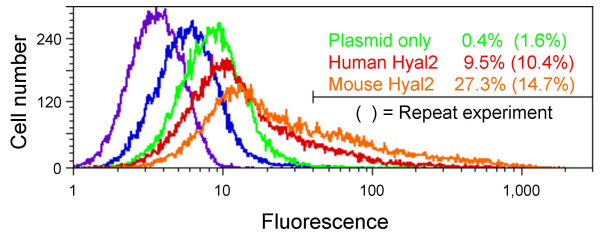
**Cell-surface mouse Hyal2 expression levels are at least as high as those of human Hyal2 following expression plasmid transfection**. Plasmids encoding amino-terminal Flag-tagged mouse or human Hyal2, or an empty plasmid as a control, were cotransfected with pSV2neo at a 10:1 ratio into NIH 3T3 cells. One day after transfection the cells were treated with trypsin and seeded in medium containing G418. Two weeks later, cell-surface Flag-tagged protein expression was measured by FACS after incubation of the cells with an anti-Flag mouse monoclonal primary antibody followed by a goat anti-mouse IgG secondary antibody. Green indicates cells transfected with control plasmid, red indicates cells transfected with Flag-tagged human Hyal2, and orange indicates cells transfected with Flag-tagged mouse Hyal2. Control analyses included Flag-tagged human Hyal2-transfected cells that were not incubated with antibody (purple) or with secondary antibody only (blue). The percentages of cells in the indicated gate were determined and are shown in the inset. The entire experiment was repeated and the repeat values are shown in parentheses.

We considered the possibility that the low JSRV receptor activity of mouse Hyal2 was simply due to poor processing of mouse Hyal2 to the cell surface in comparison to human Hyal2. To test this possibility, we generated stable cell lines containing plasmids that encoded Flag-tagged human or mouse Hyal2, or a plasmid that did not contain a eukaryotic expression cassette. The plasmids were introduced into cells by cotransfection with a plasmid encoding neomycin phosphotransferase (Neo) and by selection of the cells in G418. FACS analysis using an anti-Flag monoclonal primary antibody and a fluorescently-labeled anti mouse IgG secondary antibody showed increased Flag levels on the cells containing the Flag-tagged mouse and human plasmids in comparison to the control plasmid, with the highest levels of expression on the cells expressing the Flag-tagged mouse Hyal2 (Fig. 4). We conclude that the reason that mouse Hyal2 does not act as a receptor for JSRV is not because of poor processing of the mouse protein to the cell surface.

### Mapping of human Hyal2 amino acids important for receptor function to the bee venom hyaluronidase crystal structure indicates a potential site for Hyal2/Env interaction

**Figure 5:**
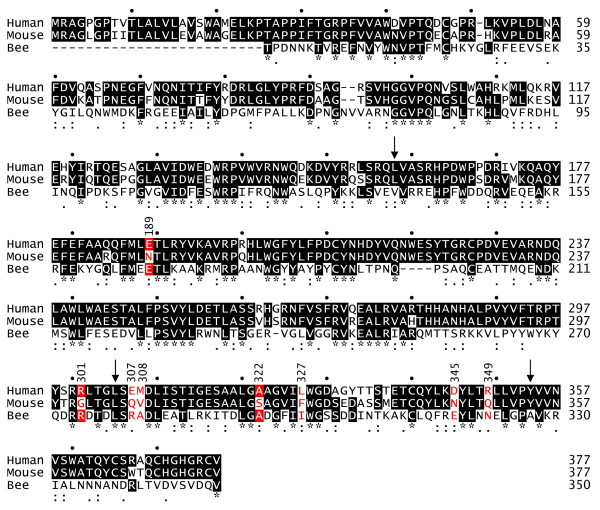
**Amino acid alignment of human Hyal2, C3H mouse Hyal2, and bee venom Hya**. Proteins were aligned by using ClustalW [23]. The sequence of the mature protein is shown for Hya [10], while amino acids 1 – 377 of the precursor forms of the Hyal2 proteins are shown. Amino acid residues colored red indicate the positions at which alteration of human to mouse Hyal2 amino acids have the largest effects on the receptor activity of human Hyal2. White letters indicate amino acids shared between at least two sequences, and black or red letters indicate unique amino acids at a given position. Amino acid identity (*), strong similarity (:), weaker similarity (.), and dissimilarity (no mark) are indicated below the sequences, numbers above the sequences refer to amino acid position in the Hyal2 sequences, and dots indicate 10 base-pair intervals in the Hyal2 sequences. Downward arrows indicate positions of junctions in the chimeric human/mouse Hyal2 chimeras.

**Figure 6:**
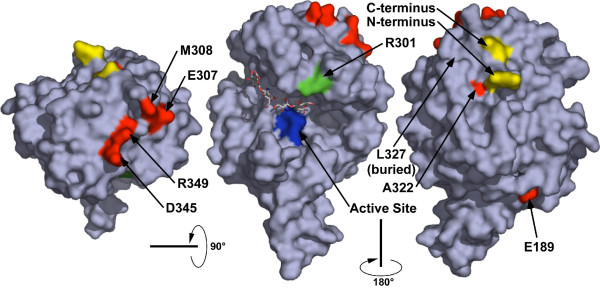
**Mapping of amino acid residues that are important for the receptor function of human Hyal2 to the bee venom Hya structure**. Amino acids of human Hyal2 were mapped to the bee venom Hya protein structure [PDB:1FCV]. Positions at which alteration of the human Hyal2 sequence to that of the mouse resulted in a decrease in receptor activity are colored red, those that resulted in an increase in receptor function are colored green. The hyaluronic acid tetramer that was present in the crystal structure and the active site of the enzyme (blue) can be seen in the middle structure. Protein structure images were generated by using PyMOL [24].

The receptor activities of human, rat and mouse Hyal2 proteins (high, low and none, respectively) correlate with their relative abilities to bind JSRV Env [8]. Assuming that Hyal2 receptor activity is primarily determined by its ability to bind Env, the positions of mutations in Hyal2 that affect receptor activity might define a binding site for Env on Hyal2. None of the 3-dimensional structures of mammalian hyaluronidase proteins have been determined, but the sequence of the hyaluronidase found in bee venom (Hya) is quite similar to those of the mammalian Hyal2 proteins (Fig. 5), and its crystal structure has been solved [10]. In an attempt to define a potential binding site for JSRV Env on Hyal2, we used the amino acid alignment shown in Fig. 5 to map the amino acid residues in human Hyal2 that are important for receptor activity to the crystal structure of bee venom hyaluronidase (Fig. 6). This crystal structure includes a hyaluronic acid tetramer (stick structure) which lies in a groove containing the enzyme active site (colored blue) (Fig. 6, middle structure).

Of the seven human Hyal2 amino acids that when mutated to the mouse sequence showed the largest negative effects (colored red in Fig. 6), 4 mapped to one surface patch on Hyal2 (E307, M308, D345, and R349) (Fig. 6, left structure), two mapped under this patch (A322 and L327) (Fig. 6, right structure), and one mapped to a distant surface site (E189) (Fig. 6, right structure). Two of the residues in the surface patch are on the same loop of a helix (E307 and M308) while the other two are on adjacent loops of another helix (D345 and R349). The L327F mutation in human Hyal2 that results in a 5-fold reduction in Hyal2 receptor activity lies just underneath this surface patch with the side chain of this amino acid pointing toward the patch, and the increased size of phenylalanine compared to leucine might cause movement of the 2 helices that compose the surface patch resulting in loss of receptor activity. It is more difficult to explain the 9-fold reduction of Hyal2 receptor activity caused by the A322S mutation, but this amino acid also maps close to the surface patch and may cause distortion of the patch. This residue is unlikely to be involved directly in Env binding, even though it has a surface localization, because additional amino acids at the N- and C- termini of Hyal2 that cannot be mapped to the Hya structural model would likely interfere with Env binding to this amino acid residue (see Fig. 6, right structure).

The human Hyal2 amino acid that when mutated to the mouse sequence showed the largest positive effect (R301, colored green in Fig. 6, middle structure) maps to the surface near the active site of Hya and could interact with Env, while E189 is distant from the proposed binding patch (Fig. 6, right structure). Thus a plausible binding site for Env that involves many of the important amino acid residues in human Hyal2 is predicted by this analysis.

### Comparison of mammalian Hyal2 sequences show strong pressure to conserve the protein sequence but no evidence for selective pressure to avoid JSRV infection

Viruses bearing the JSRV Env protein can infect cells from multiple mammalian species, and we considered the possibility that there might be selection during the evolution of mammals for Hyal2 variants that cannot act as receptors for these viruses. Evidence for such selection has been found for other proteins involved in virus defense, including APOBEC3G and TRIM5α [11,12]. The location of positively selected amino acid variations might provide additional data regarding the Env binding site on Hyal2. In particular, we previously found that bovine Hyal2 acts as a weak receptor for JSRV and ENTV compared to sheep or human Hyal2, but bovine Hyal2 is much more closely related to sheep Hyal2 than to human Hyal2, suggesting that there might be specific selection in cattle for resistance to transmission of JSRV or ENTV from sheep and goats [6].

Positive selection for alteration in protein function can be detected by measuring the rate of non-synonymous (Ka) versus synonymous (Ks) nucleotide changes in DNA during species evolution. Ka/Ks = 1 indicates a lack of selection for or against the protein sequence, Ka/Ks < 1 indicates negative selection, or selection for amino acid conservation, and Ka/Ks > 1 indicates positive selection for protein alteration. We examined the Ka/Ks ratios for pairwise combinations of Hyal2 proteins from sheep, cattle, pig, dog, human, mouse, and rat (see Additional files 1, 2, 3 for DNA sequences used and amino acid alignment) by using the K-estimator 6.1 program created by JM Comeron [13]. The overall Ka/Ks values ranged from 0.11 to 0.23, indicating strong selection for amino acid conservation in Hyal2 proteins from these species. We also analyzed Ka/Ks ratios using a sliding window approach to detect particular regions of Hyal2 that might be under positive (diversifying) selection, but again found no evidence for such selection (data not shown).

Strong selection for amino acid conservation can mask small numbers of amino acids undergoing positive selection for altered protein function, so we looked more closely for amino acid selection at the single amino acid level. Analysis for selection at individual amino acid sites using the Single Likelihood Ancestor Counting (SLAC) program [14-16] identified 48 negatively-selected (conserved) sites and no positively-selected sites at the 0.1 level of significance. Further analysis using the Random Effects (REL) program [14-16], that provides a less conservative analysis for selection at individual amino acid sites, found 35 negatively-selected (conserved) sites and no positively-selected sites at the default program significance setting of 50. Together these results provide strong evidence for conservation of Hyal2 protein sequence but no evidence for positive selection to resist virus infection.

## Discussion

We have found that the difference in activity of human and mouse Hyal2 as entry receptors for viruses bearing the JSRV Env are determined by multiple amino acid differences between the two proteins. This is in contrast to results of a similar analysis of the activity of human and mouse CAT1 as entry receptors for ecotropic murine leukemia viruses. In the latter case, mouse CAT1 is active while human CAT1 is inactive as a receptor. Substitution of only one amino acid in the mouse receptor with the corresponding amino acid from the human receptor was enough to abrogate receptor activity, and substitution of 2 amino acid residues in human CAT1 with those of mouse CAT1 could convert human CAT1 into a functional receptor [17]. In other examples, amino acid changes resulting in receptor glycosylation were found to be responsible for the lack of receptor activity in receptor orthologs from cells that were resistant to virus infection [18,19]. In the case of mouse and human Hyal2 there are no predicted N- or O-linked glycosylation sites in the middle region of these proteins that we found was responsible for the difference in receptor activity, indicating that the difference in receptor activity is not due to a difference in glycosylation.

We used amino-terminal Flag tags to show that the mouse and human Hyal2 proteins were both processed to the cell surface. Levels of mouse Hyal2 were somewhat higher than those of human Hyal2, indicating that the lack of mouse Hyal2 receptor activity was not due to poor processing of the protein to the cell surface. We did not perform this analysis for the mouse/human Hyal2 chimeras or for the mutant Hyal2 proteins, and some proportion of reduced receptor activity we detected in some constructs could be due to improper folding and or processing of the proteins to the cell surface. We attempted to measure cell-surface processing of mouse and human Hyal2 at 2 days after Hyal2 plasmid transfection, at the same time we assay for JSRV vector transduction, but the cell-surface Flag signal was too low to allow reliable quantitation. Selection for cells expressing the transfected plasmid was required for Flag detection, and even then the signal was heterogeneous (Fig. 4). In retrospect, a better way to perform these studies might have been to Flag-tag all of the Hyal2 constructs and insert these cDNAs into a retroviral vector, to generate virus using packaging cell lines, to transduce the target cells at a multiplicity of infection such that each cell received one vector copy, and to select the cells for the presence of the vector. These selected populations would then stably express the Hyal2 constructs and could then have been assayed for susceptibility to JSRV vector transduction and for cell-surface Flag expression to provide more accurate data on cell-surface protein processing and receptor activity. Regardless, results presented here allow us to draw some strong conclusions about the determinants of Hyal2 receptor function. For example, if any of the human Hyal2 mutants containing the corresponding mouse Hyal2 residues had no activity, it would have been important to show that this result was not due simply to a processing defect. However, all mutants had activity well above the mouse Hyal2 level, and were thus functional to some extent, allowing us to conclude that multiple amino acid differences contribute to the low activity of mouse Hyal2 in comparison to that of human Hyal2.

We have mapped four of the amino acid residues that are important for human Hyal2 function as a virus receptor to a surface patch on the bee venom Hya crystal structure, and two others map to positions underneath this patch and could influence the structure of the patch. We hypothesize that this patch represents the binding site for Env, but it is also possible that this site is required for Hyal2 binding to another protein involved in virus entry. Furthermore, this structural extrapolation to the bee venom hyaluronidase may not be entirely valid and determination of a crystal structure for mouse or human Hyal2 is important to confirm these predictions. A soluble form of human Hyal2 has been made that can bind tightly to JSRV Env, as measured by surface plasmon resonance analysis, and that can block JSRV vector transduction by binding to JSRV vector virions [3]. We are currently working to crystallize this protein to allow more definitive analysis of results presented here.

## Conclusion

We show that the greatly reduced receptor activity of mouse Hyal2 in comparison to that of human Hyal2 is determined by multiple amino acid changes acting in concert, and that no one amino acid change blocks infection. However, the most important amino acids map to a small patch on a predicted 3-dimensional Hyal2 structure which we hypothesize is the binding site for Env.

## Methods

### Cell culture and virus production

NIH 3T3 (TK^-^) Swiss mouse embryo fibroblasts [20], 208F Fischer rat embryo fibroblasts [21], and PJ4/LAPSN vector-producing cells [4] were grown in Dulbecco's modified Eagle medium (DMEM) with high glucose (4.5 g/L) and 7% fetal bovine serum at 37°C in a 10% CO_2_-air atmosphere at 100% relative humidity. PJ4/LAPSN cells produce virus containing the LAPSN retroviral vector [22] that encodes human placental alkaline phosphatase (AP) and neomycin phosphotransferase (Neo). Virus produced by PJ4/LAPSN cells contain Moloney murine leukemia virus Gag-Pol proteins and have a JSRV Env protein coat. LAPSN(PJ4) vector was produced by feeding confluent PJ4/LAPSN cells and harvesting the medium 12 to 24 h later. Virus-containing medium was centrifuged at 3,000 × g for 5 min to remove cells and debris and was frozen at -70°C until use.

### Plasmid construction

All Hyal2 plasmids tested were constructed in either the pFLAG-CMV-1 mammalian expression vector (Sigma-Aldrich, Saint Louis, MO) or the pCR3.1 TOPO eukaryotic expression vector (Invitrogen, Carlsbad, CA). Each of the human Hyal2 amino or carboxy terminus deleted plasmids was cloned into the *Eco*RI site of the pFLAG-CMV-1 vector after PCR amplification of the desired coding regions. The human and mouse Hyal2 mutants containing single, double or triple amino acid substitutions were created by using the QuikChange Site-Directed Mutagenesis Kit (Stratagene, La Jolla, CA) following recommended protocols. Human and mouse Hyal2 cDNAs share two common unique restriction sites, *Pfl*MI (located at codon 158), and *Bsa*AI (located at codon 354). The mouse Hyal2 cDNA contains 3 *Blp*I restriction sites (located near codons 305, 321, and 413), which are completely absent in human Hyal2. By performing site-directed mutagenesis without altering the encoded amino acids, a *Blp*I site near codon 305 was created in human Hyal2 and the *Blp*I site near codon 321 was eliminated in mouse Hyal2. Both the human and mouse Hyal2 can therefore be divided into 4 domains by the 3 restriction sites: *Pfl*MI (codon 158), *Blp*I (codon 305), and *Bsa*AI (codon 354). The chimeric mouse/human Hyal2 plasmids were obtained by exchanging restriction fragments between human and mouse Hyal2. The Hyal2 coding regions of all plasmids were sequenced after construction to confirm the expected sequences.

### Receptor activity assay

For assay, NIH 3T3 mouse cells or 208F rat cells were seeded at 2.5 × 10^5 ^per well (d = 3.5 cm) of 6-well plates. The next day the cells were transfected with 4.5 μg of the test Hyal2 plasmid plus 0.5 μg of a plasmid encoding β-galactosidase (β-gal) per well by calcium phosphate coprecipitation as previously described [8]. One day after transfection the cells were trypsinized and divided 1:6 into 6-well plates. Two days after transfection the cells were exposed to serial dilutions of LAPSN(PJ4) vector in the presence of 4 μg/ml Polybrene. Four days after transfection the cells exposed to the LAPSN(PJ4) vector were stained for AP to determine the vector titer and cells not exposed to the vector were stained for β-gal expression to evaluate transfection efficiency for each plasmid. Experiments for which β-gal staining was low or variable were not included in the results. In each experiment the LAPSN(PJ4) vector titer was measured on cells transfected with an unmodified human Hyal2 expression plasmid, and results for the test plasmids are expressed as a percentage of that for the human Hyal2 plasmid (typically 10^4 ^to 3 × 10^4 ^AP^+ ^focus-forming units (FFU) per ml following transfection of NIH 3T3 mouse cells and 10^3 ^to 2 × 10^3 ^FFU/ml following transfection of 208F rat cells with the human Hyal2 plasmid). Several Hyal2 constructs were tested in both mouse and rat cells with similar results, but most of the receptor activity assays shown were performed only in the mouse cells. Results are means of at least two independent experiments.

### FACS analysis

Cell-surface expression of amino-terminal Flag-tagged Hyal2 proteins was evaluated by FACS analysis after incubation of cells with anti-Flag mouse monoclonal primary antibody followed by incubation in goat anti-mouse IgG secondary antibody. Propidium iodide was added to the cells before analysis and only cells that excluded the dye (live cells) were included in the analysis. 30,000 live cells were analyzed per sample.

## Competing interests

The author(s) declare that they have no competing interests.

## Authors' contributions

FMD designed and made all of the plasmid constructs, CD performed some initial transfection analyses, MIL helped design the plasmid constructs and the experiments, and ADM assayed the function of the wild-type, chimeric, and mutant receptors, analyzed the data, and drafted the manuscript. All authors read and approved the final manuscript.

## Supplementary Material

Additional File 1 **"Hyal2 DNA sequences.fasta"**. Bovine [GenBank:AF411973.1], ovine [GenBank:AF411974.1], pig [GenBank:AY497544.1], dog [GenBank:XM_541876.2], human [GenBank:U09577.1], C3H mouse [GenBank:AF302843.1] (same as NIH Swiss mouse), Czech II mouse [GenBank:AF302844.1, and rat [GenBank:AF535141.1] Hyal2 DNA sequences in Fasta format. Click here for file

Additional File 2 **"Hyal2 DNA sequences.phy"**. DNA sequence alignment of bovine, ovine, pig, dog, human, mouse (2 alleles), and rat Hyal2 DNA sequences in PHYLIP format made by using ClustalW [23] and suitable for use with K-estimator [13] software or for upload into Fast Positive and Negative Selection Detection website [16]. Click here for file

Additional File 3 **"Hyal2 protein alignment.pdf"**. Alignment of bovine, ovine, pig, dog, human, mouse (2 alleles), and rat Hyal2 made by using ClustalW [23] software. Click here for file
