# Supplementary material for: Amino acid residues that are important for Hyal2 function as a receptor for jaagsiekte sheep retrovirus
Source: Retrovirology. 2005 Sep 28;2:59. doi: 10.1186/1742-4690-2-59 (PMC1262777; doi:10.1186/1742-4690-2-59)
Supplement: Additional File 3 — "Hyal2 protein alignment.pdf". Alignment of bovine, ovine, pig, dog, human, mouse (2 alleles), and rat Hyal2 made by using ClustalW [23] software. [file 1742-4690-2-59-S3.pdf]

CLUSTAL W (1.75) multiple sequence alignment

```
bHyal2          MWTGLGPAVTLALVLVVAWATELKPTAPPIFTGRPFVVAWDVPTQDCGPRHKMPLDPKDM 60
oHyal2          MWTGLGPAVTLALVLVVAWATELKPTAPPIFTGRPFVVAWDVPTQDCGPRHKMPLDPKDM 60
pHyal2          MWAGLGPTVTLALVLAVAWATELKPTAPPIFTGRPFVVAWDVPTQDCGPRHKVPLDPKDM 60
dHyal2          MWAGLGPAVTLALV-VVAWAAQLKPTAPPIFTGRPFVVAWDVPTQDCGPRLKVPLD---L 56
hHyal2          MRAGPGPTVTLALVLAVSWAMELKPTAPPIFTGRPFVVAWDVPTQDCGPRLKVPLD---L 57
mHyal2          MRAGLGPIITLALVLEVAWAGELKPTAPPIFTGRPFVVAWNVPTQECAPRHKVPLD---L 57
mHyal2a         MRAGLGPIITLALVLEVAWAGELKPTAPPIFTGRPFVVAWNVPTQECAPRHKVPLD---L 57
rHyal2          MRAGLGPIITLALVLEVAWASELKPTAPPIFTGRPFVVAWNVPTQECAPRHKVPLD---L 57
                * :* ** :*****  *:** :******************:****:*.** *:***   :

bHyal2          KAFDVQASPNEGFVNQNITIFYRDRLGMYPHFNSVGRSVHGGVPQNGSLWVHLEMLKGHV 120
oHyal2          KAFDVQASPNEGFVNQNITIFYRDRLGMYPHFNSVGRSVHGGVPQNGSLWVHLEMLKGHV 120
pHyal2          KAFDVQASPNEGFVNQNITIFYRDRLGMYPHFDSVGRSVHGGVPQNGSLWVHLKMLKGHV 120
dHyal2          KAFDVQASPNEGFVNQNITIFYHDRLGLYPRFSSVGRSVHGGVPQNGSLWAHLKMLQEHV 116
hHyal2          NAFDVQASPNEGFVNQNITIFYRDRLGLYPRFDSAGRSVHGGVPQNVSLWAHRKMLQKRV 117
mHyal2          RAFDVKATPNEGFFNQNITTFYYDRLGLYPRFDAAGTSVHGGVPQNGSLCAHLPMLKESV 117
mHyal2a         RAFDVKATPNEGFFNQNITTFYYDRLGLYPRFDAAGTSVHGGVPQNGSLCAHLPMLKESV 117
rHyal2          RAFDVEATPNEGFFNQNITTFYYDRLGLYPRFDAAGMSVHGGVPQNGSLCAHLPMLKEAV 117
                .****:*:*****.***** ** ****:**:*.:.* ********* ** .*  **:  *

bHyal2          EHYIRTQEPAGLAVIDWEDWRPVWVRNWQDKDVYRRLSRHLVAIRHPDWPPERVAKEAQY 180
oHyal2          EHYIRTQEPAGLAVIDWEDWRPVWVRNWQDKDVYRRLSRQLVASHHPDWPPERIVKEAQY 180
pHyal2          EHYIRTQEPAGLAVIDWEDWRPVWVRNWQDKDVYRRLSRQLVASRHPDWPPDRVVKQAQY 180
dHyal2          EHYIRSQEPAGLAVIDWEDWRPVWVRNWQDKDIYRQSSRQLVAVRHPDWPADRVVKQAQY 176
hHyal2          EHYIRTQESAGLAVIDWEDWRPVWVRNWQDKDVYRRLSRQLVASRHPDWPPDRIVKQAQY 177
mHyal2          ERYIQTQEPGGLAVIDWEEWRPVWVRNWQEKDVYRQSSRQLVASRHPDWPSDRVMKQAQY 177
mHyal2a         ERYIQTQEPGGLAVIDWEEWRPVWVRNWQEKDVYRQSSRQLVASRHPDWPSDRVMKQAQY 177
rHyal2          ERYIQTQEPAGLAVIDWEEWRPVWVRNWQEKDVYRQSSRQLVASRHPDWPSDRIVKQAQY 177
                *:**::**..********:**********:**:**: **:*** :*****.:*: *:***

bHyal2          EFEFAARQFMLETLRFVKAFRPRHLWGFYLFPDCYNHDYVQNWETYTGRCPDVEVSRNDQ 240
oHyal2          EFEFAARQFMLETLRFVKAFRPRHLWGFYLFPDCYNHDYVQNWETYTGRCPDVEVSRNDQ 240
pHyal2          EFEFAARQFMLETLRFVKAFRPQHLWGFYLFPDCYNHDYVQNWETYTGRCPDVEVSRNDQ 240
dHyal2          EFEFAARQFMLETLRFVKAVRPRHLWGFYLFPDCYNHDYVQNWETYTGRCPDVEVSRNDQ 236
hHyal2          EFEFAAQQFMLETLRYVKAVRPRHLWGFYLFPDCYNHDYVQNWESYTGRCPDVEVARNDQ 237
mHyal2          EFEFAARQFMLNTLRYVKAVRPQHLWGFYLFPDCYNHDYVQNWESYTGRCPDVEVARNDQ 237
mHyal2a         EFEFAARQFMLNTLRYVKAVRPQHLWGFYLFPDCYNHDYVQNWESYTGRCPDVEVARNDQ 237
rHyal2          EFEFAARQFMLNTLRYVKAVRPQHLWGFYLFPDCYNHDYVQNWDSYTGRCPDVEVARNDQ 237
                ******:****:***:***.**:********************::**********:****

bHyal2          LAWLWAESTALFPSVYLEETLASSTHGRNFVSFRVQEALRVADVHHANHALPVYVFTRPT 300
oHyal2          LSWLWAESTALFPSVYLEETLASSTHGRNFVSFRVQEALRVADVHHANHALPVYVFTRPT 300
pHyal2          LAWLWAESTALFPSVYLEETLASSTHGRNFVSFRVQEALRVAHTHHANHALPVYVFTRPT 300
dHyal2          LAWLWAESTALFPSVYLDETLASSTHGRNFVSFRVQEALRVAHTHHANHALPVYVFTRPT 296
hHyal2          LAWLWAESTALFPSVYLDETLASSRHGRNFVSFRVQEALRVARTHHANHALPVYVFTRPT 297
mHyal2          LAWLWAESTALFPSVYLDETLASSVHSRNFVSFRVREALRVAHTHHANHALPVYVFTRPT 297
mHyal2a         LAWLWAESTALFPSVYLDETLASSVHSRNFVSFRVREALRVAHTHHANHALPVYVFTRPT 297
rHyal2          LAWLWAESTALFPSVYLDETLASSKHSRNFVSFRVQEALRVAHTHHANHALPVYVFTRPT 297
                *:***************:****** *.********:****** .****************

bHyal2          YSRGLTGLSEMDLISTIGESAALGAAGVILWGDAGFTTSNETCRRLKDYLTRSLVPYVVN 360
oHyal2          YSRGLTGLSEMDLISTIGESAALGAAGVILWGDAGFTTSNETCRRLKDYLTRSLVPYVVN 360
pHyal2          YSRGLTGLSEMDLISTIGESAALGAAGVILWGDAGYTTSMETCQYLKDYLTRLLVPYVVN 360
dHyal2          YSRRLTGLSEMDLISTIGESAALGAAGVILWGDAGYTTSTETCQYLKDYLKRLLVPYVVN 356
hHyal2          YSRRLTGLSEMDLISTIGESAALGAAGVILWGDAGYTTSTETCQYLKDYLTRLLVPYVVN 357
mHyal2          YTRGLTGLSQVDLISTIGESAALGSAGVIFWGDSEDASSMETCQYLKNYLTQLLVPYVVN 357
mHyal2a         YTRGLTGLSQVDLISTIGESAALGSAGVIFWGDSEDASSMETCQYLKNYLTQLLVPYIVN 357
rHyal2          YTRGLTELSQMDLISTIGESAALGSAGVIFWGDSVYASSMENCQNLKKYLTQTLVPYIVN 357
                *:* ** **::*************:****:***:  ::* *.*: **.**.: ****:**
```

```
bHyal2          VSWAAQYCSWAQCHGHGRCVRRDPNAHTFLHLSASSFRLVPSHAPDEPRLRPEGELSWAD 420
oHyal2          VSWAAQYCSWAQCHGHGRCVRRDPNAHTFLHLSASSFRLVPSHAPDEPRLRPEGELSWAD 420
pHyal2          VSWAAQYCSWAQCHGHGRCVRRDPSANIFLHLSASSFRLVASHAPGEPQLRPEGELSWAD 420
dHyal2          VSWAAQYCSWAQCHGHGRCVRRDPSANTFLHLSASSFRLVPSHVPGEPQLRPEGELSWAD 416
hHyal2          VSWATQYCSRAQCHGHGRCVRRNPSASTFLHLSTNSFRLVPGHAPGEPQLRPVGELSWAD 417
mHyal2          VSWATQYCSWTQCHGHGRCVRRNPSANTFLHLNASSFRLVPGHTPSEPQLRPEGQLSEAD 417
mHyal2a         VSWATQYCSWTQCHGHGRCVRRNPSANTFLHLNASSFRLVPGHTPSEPQLRPEGQLSEAD 417
rHyal2          VSWATQYCSWTQCHGHGRCVRRNPSASTFLHLSPSSFRLVPGRTPSEPQLRPEGELSEDD 417
                ****:**** :***********:*.*  ****...*****..:.*.**:*** *:**  *

bHyal2          RNHLQMHFRCQCYLGWGGEQCQWDRRRAAGGASGAWAGSHLTGLLAVAVLAFT--- 473
oHyal2          RNHLQTHFRCQCYLGWGGEQCQWDRRRAAGGASGAWAGSHLTGLLAVAVLAFTWTS 476
pHyal2          RNHLQTHFRCQCYVGWGGEQCQRDLRRAAGDASRAWAGSHLTSLLALAALAYPGTL 476
dHyal2          LNHLQTHFRCQCYLGWGGEQCQWDHTRAAGGARGAWAGSHLTGPLAVAALVLTWTS 472
hHyal2          IDHLQTHFRCQCYLGWSGEQCQWDHRQAAGGASEAWAGSHLTSLLALAALAFTWTL 473
mHyal2          LNYLQKHFRCQCYLGWGGEQCQRNYKGAAGNASRAWAGSHLTSLLGLVAVALTWTL 473
mHyal2a         LNYLQKHFRCQCYLGWGGEQCQRNYKGAAGNASRAWAGSHLTSLLGLVAVALTWTL 473
rHyal2          LSYLQMHFRCHCYLGWGGEQCQWNHKRAAGDASRAWAGAHLASLLGLVAMTLTWTL 473
                 .:** ****:**:**.***** :   ***.*  ****:**:. *.:..:. .
```
